# Supplementary material for: Freely accessible ready to use global infrastructure for SARS-CoV-2 monitoring
Source: bioRxiv. 2021 Mar 25:2021.03.25.437046. Preprint. [Version 1] doi: 10.1101/2021.03.25.437046 (PMC8010728; doi:10.1101/2021.03.25.437046)

bioRxiv preprint doi: https://doi.org/10.1101/2021.03.25.437046; this version posted March 25, 2021. The copyright holder for this preprint (which was not certified by peer review) is the author/funder, who has granted bioRxiv a license to display the preprint in perpetuity. It is made available under aCC-BY 4.0 International license.

Table S1. Parameters used for comparing performance of allelic-variant callers

Caller	Command line	Figure 2 label
mutect2	--mitochondria-mode true	m
mutect2	default	m_noM
mutect2	--mitochondria-mode true<br>--f1r2-max-depth 1000000	m_md_inf
mutect2	--mitochondria-mode true<br>--f1r2-max-depth 1000000<br>-max-af 1	m_md_inf_max_af1
freebayes	--haplotype-length 0<br>--min-alternate-fraction 0.001<br>--min-alternate-count 1<br>--pooled-continuous --ploidy 1	hl-0_maf-001_pc
freebayes	-min-alternate-fraction 0.001<br>--pooled-continuous --ploidy 1	maf-001_pc
lofreq	--no-default-filter	nf
lofreq	default	def

bioRxiv preprint doi: https://doi.org/10.1101/2021.03.25.437046; this version posted March 25, 2021. The copyright holder for this preprint (which was not certified by peer review) is the author/funder, who has granted bioRxiv a license to display the preprint in perpetuity. It is made available under aCC-BY 4.0 International license.

Table S2. Links to Galaxy histories containing COG-UK reanalysis results. A continuously updated list can be accessed https://github.com/galaxyproject/SARS-CoV-2/tree/master/data/cog-uk-tracking. These datasets will also be available from the Viral Beacon project at (https://covid19beacon.crg.eu/).

Galaxy histories	contained ENA IDs	AV history (with VCF of all called variants)	variant report history (with tabular reports of all variants)
COG-UK NT1659949O	ERR5379175-ERR5379540	https://usegalaxy.eu/histories/view?id=a70e27d468a29519	-
COG-UK NT1659948N	ERR5378946-ERR5379174	https://usegalaxy.eu/histories/view?id=d67968ed5fe74586	https://usegalaxy.eu/histories/view?id=762cd32fdd59b3ef
COG-UK NT1659945K	ERR5352103-ERR5352455	https://usegalaxy.eu/histories/view?id=97050f0c7217eb53	https://usegalaxy.eu/histories/view?id=1bf724bcb1a6bce2
COG-UK NT1659944J	ERR5351745-ERR5352102	https://usegalaxy.eu/histories/view?id=c5156eda4b3c5e24	https://usegalaxy.eu/histories/view?id=55823ef9c77a410d
COG-UK NT1659891N	ERR5378314-ERR5378575	https://usegalaxy.eu/histories/view?id=c25d0f298ac2ef34	https://usegalaxy.eu/histories/view?id=d30fa7c11c245bd7
COG-UK NT1659885P	ERR5352718-ERR5375533	https://usegalaxy.eu/histories/view?id=8f829af16c834443	https://usegalaxy.eu/histories/view?id=df02b799e2c42a7c
COG-UK NT1660206U	ERR5382276-ERR5382582	https://usegalaxy.eu/histories/view?id=484f288640bdbbc4	https://usegalaxy.eu/histories/view?id=612b0916712b673a
COG-UK NT1660207V	ERR5382583-ERR5382926	https://usegalaxy.eu/histories/view?id=23191ca01f1766fd	https://usegalaxy.eu/histories/view?id=1c870e4766af57fa
COG-UK NT1660170C	ERR5379541-ERR5379801	https://usegalaxy.eu/histories/view?id=216df75b20548363	https://usegalaxy.eu/histories/view?id=996170468f48ad5e
COG-UK NT1660168I	ERR5380719-ERR5381079	https://usegalaxy.eu/histories/view?id=929888a678e25678	https://usegalaxy.eu/histories/view?id=a5371f3d76143ee6
COG-UK NT1660200O	ERR5380509-ERR5380718	https://usegalaxy.eu/histories/view?id=5d19cd66540f3b4f	https://usegalaxy.eu/histories/view?id=2d5595a60fe335b8
COG-UK NT1660185J	ERR5381350-ERR5381634	https://usegalaxy.eu/histories/view?id=761b945d604a584c	https://usegalaxy.eu/histories/view?id=0bb2222cb26f62fb
COG-UK NT1660171D	ERR5379802-ERR5380132	https://usegalaxy.eu/histories/view?id=ee5d68ddc0c16766	https://usegalaxy.eu/histories/view?id=b7435d647332c545

bioRxiv preprint doi: https://doi.org/10.1101/2021.03.25.437046; this version posted March 25, 2021. The copyright holder for this preprint (which was not certified by peer review) is the author/funder, who has granted bioRxiv a license to display the preprint in perpetuity. It is made available under aCC-BY 4.0 International license.

COG-UK NT1660086H	ERR5378576-ERR5378945	https://usegalaxy.eu/histories/view?id=bdfae26e713d1461	https://usegalaxy.eu/histories/view?id=7586ad8f459dfbdc
COG-UK NT1660169J	ERR5381080-ERR5381349	https://usegalaxy.eu/histories/view?id=02dd0e471db55821	https://usegalaxy.eu/histories/view?id=22410c20518125b3
COG-UK NT1660201P	ERR5380133-ERR5380314	https://usegalaxy.eu/histories/view?id=f584aa738ccbde76	-
COG-UK NT1660199P	ERR5381954-ERR5382275	https://usegalaxy.eu/histories/view?id=ee1e4a8c859879dd	-
COG-UK NT1660186K	ERR5381635-ERR5381953	https://usegalaxy.eu/histories/view?id=9dab59078524857b	-

bioRxiv preprint doi: https://doi.org/10.1101/2021.03.25.437046; this version posted March 25, 2021. The copyright holder for this preprint (which was not certified by peer review) is the author/funder, who has granted bioRxiv a license to display the preprint in perpetuity. It is made available under aCC-BY 4.0 International license.

Figure S1. Observed (red) versus predicted (orange) counts of samples sharing *N=0, 1, 2, …* varinants as a function of allelic-variant number for each dataset. The intersection of the lines gives the cutoff that was applied to each dataset.

"Boston" (https://covid19.galaxyproject.org/genomics/interactive_images/thresholding_Boston.html)

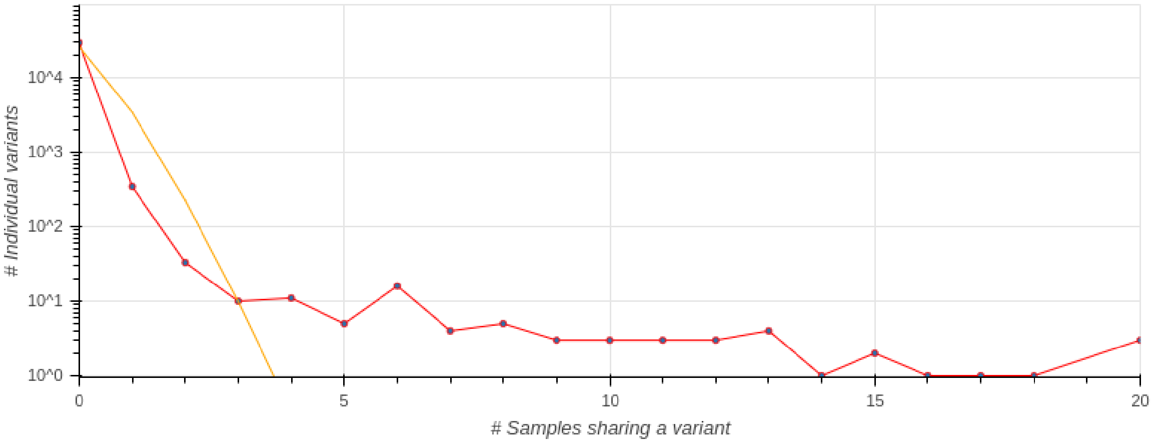

"COG-Pre" (https://covid19.galaxyproject.org/genomics/interactive_images/thresholding_COG-Pre.html)

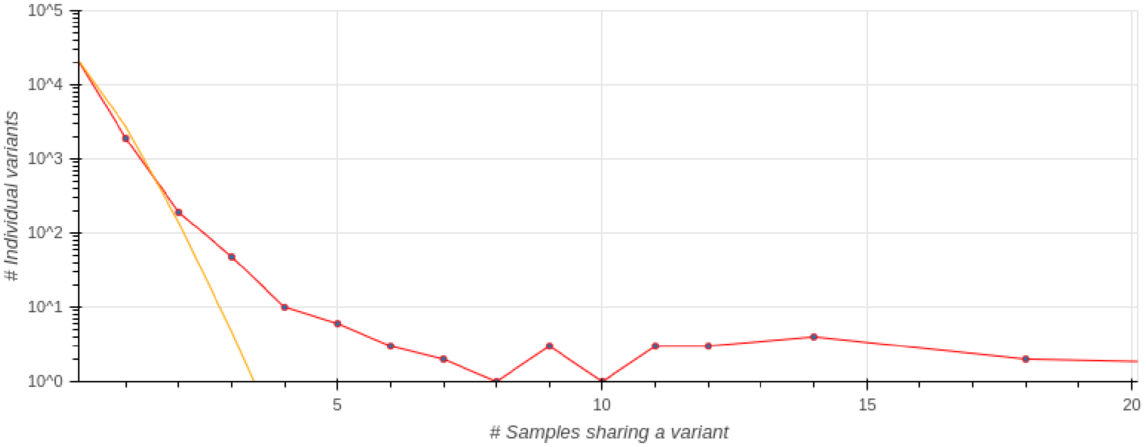

"COG-Post" (https://covid19.galaxyproject.org/genomics/interactive_images/thresholding_COG-Post.html)

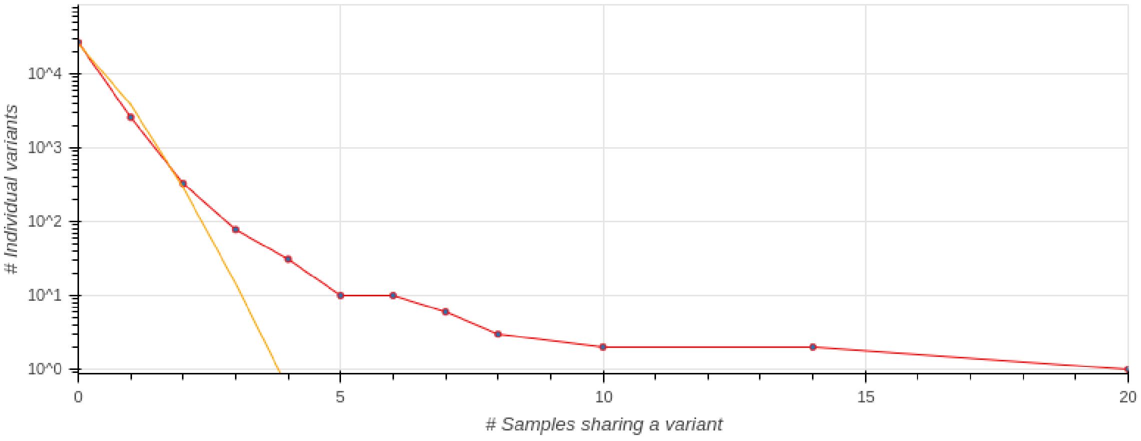

bioRxiv preprint doi: https://doi.org/10.1101/2021.03.25.437046; this version posted March 25, 2021. The copyright holder for this preprint (which was not certified by peer review) is the author/funder, who has granted bioRxiv a license to display the preprint in perpetuity. It is made available under aCC-BY 4.0 International license.

Figure S2. Distribution of allele frequencies for different types of substitutions.

"Boston" (https://covid19.galaxyproject.org/genomics/interactive_images/af_histogram_Boston.html)

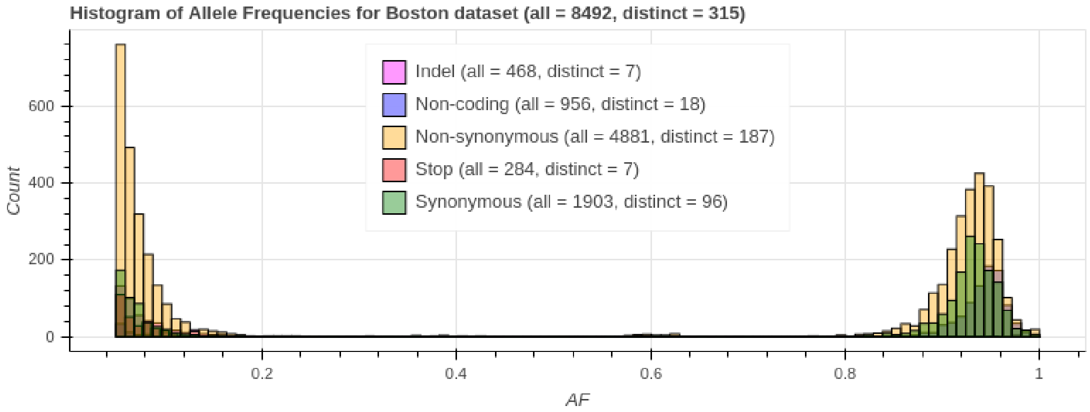

"COG-Pre" (https://covid19.galaxyproject.org/genomics/interactive_images/af_histogram_COG-Pre.html)

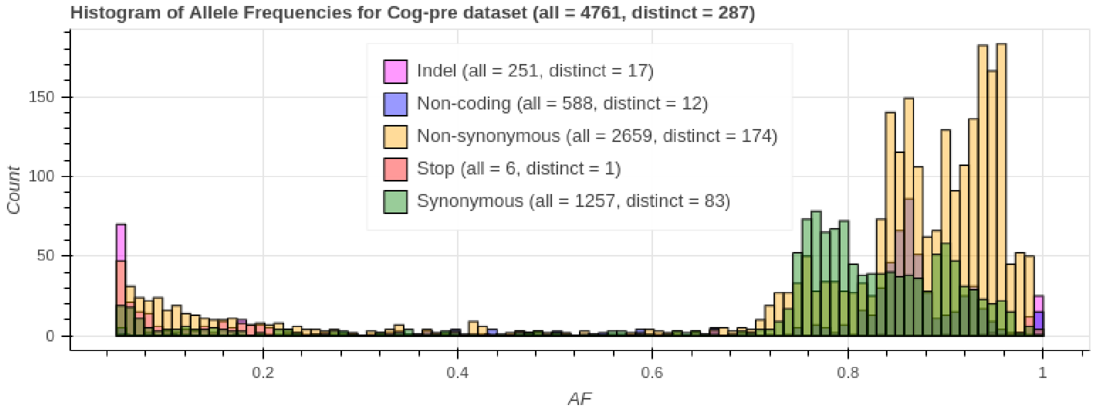

"COG-Post" (https://covid19.galaxyproject.org/genomics/interactive_images/af_histogram_COG-Post.html)

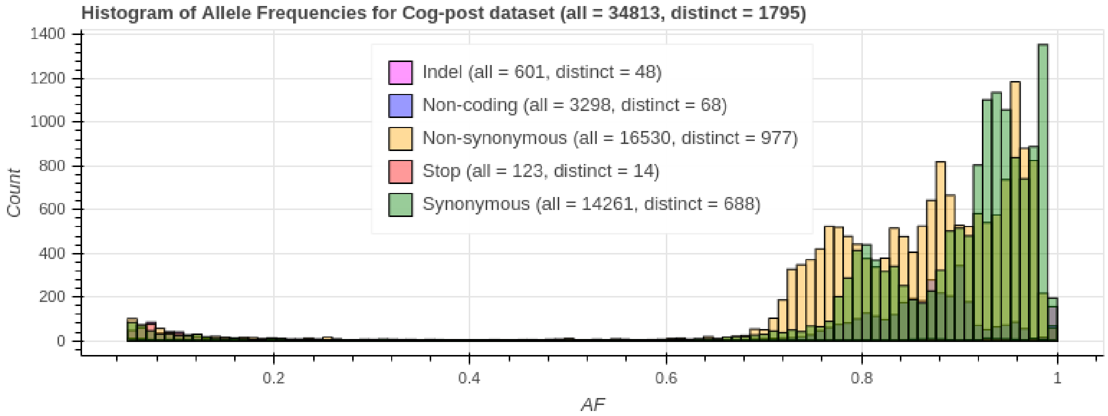

bioRxiv preprint doi: https://doi.org/10.1101/2021.03.25.437046; this version posted March 25, 2021. The copyright holder for this preprint (which was not certified by peer review) is the author/funder, who has granted bioRxiv a license to display the preprint in perpetuity. It is made available under aCC-BY 4.0 International license.

Figure S3. Types and counts of single nucleotide substitutions.

"Boston" (https://covid19.galaxyproject.org/genomics/interactive_images/substitutions_types_Boston.html)

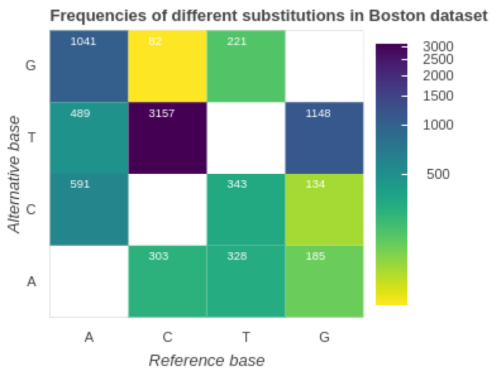

"COG-Pre" (https://covid19.galaxyproject.org/genomics/interactive_images/substitutions_types_COG-Pre.html)

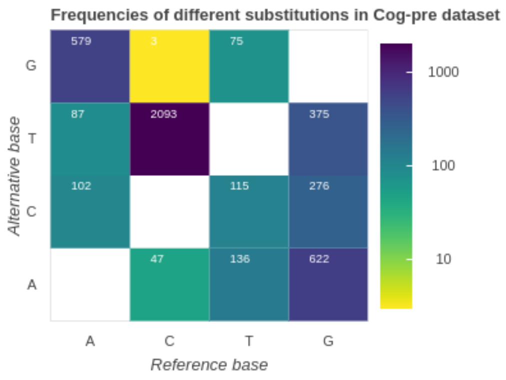

"COG-Post" (https://covid19.galaxyproject.org/genomics/interactive_images/substitutions_types_COG-Post.html)

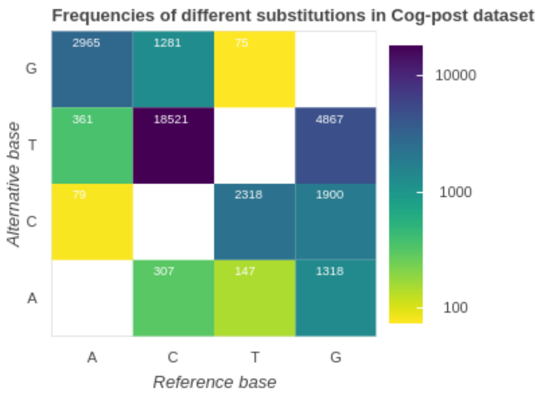

bioRxiv preprint doi: https://doi.org/10.1101/2021.03.25.437046; this version posted March 25, 2021. The copyright holder for this preprint (which was not certified by peer review) is the author/funder, who has granted bioRxiv a license to display the preprint in perpetuity. It is made available under aCC-BY 4.0 International license.

Figure S4. Relationship between population frequency and alternative allele frequency in the three datasets. CoV = coefficient of variation. PF = population frequency (e.g., how many samples in the dataset share a given variant). Points with high CoV have large spread of allele frequencies.

"Boston" https://covid19.galaxyproject.org/genomics/interactive_images/cov_vs_pf_boston.html

"COG-Pre" https://covid19.galaxyproject.org/genomics/interactive_images/cov_vs_pf_cog-pre.html

"COG-Post" https://covid19.galaxyproject.org/genomics/interactive_images/cov_vs_pf_cog-post.html

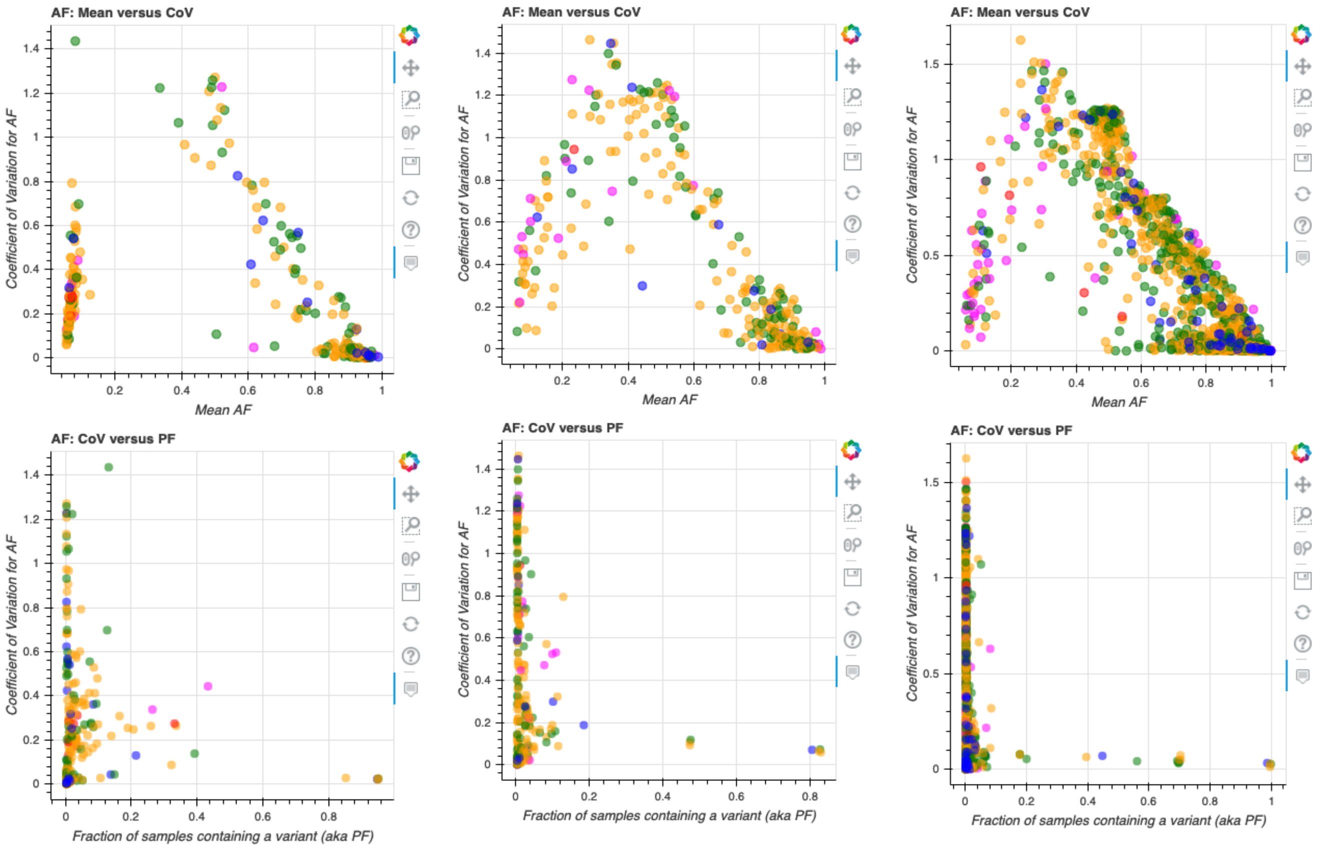

bioRxiv preprint doi: https://doi.org/10.1101/2021.03.25.437046; this version posted March 25, 2021. The copyright holder for this preprint (which was not certified by peer review) is the author/funder, who has granted bioRxiv a license to display the preprint in perpetuity. It is made available under aCC-BY 4.0 International license.

Figure S5. Distribution of allelic variants across the genome. Height of each bar is proportional to the coefficient of variation for alternative allele frequency.

"Boston" (https://covid19.galaxyproject.org/genomics/interactive_images/genomic_dist_boston.html)

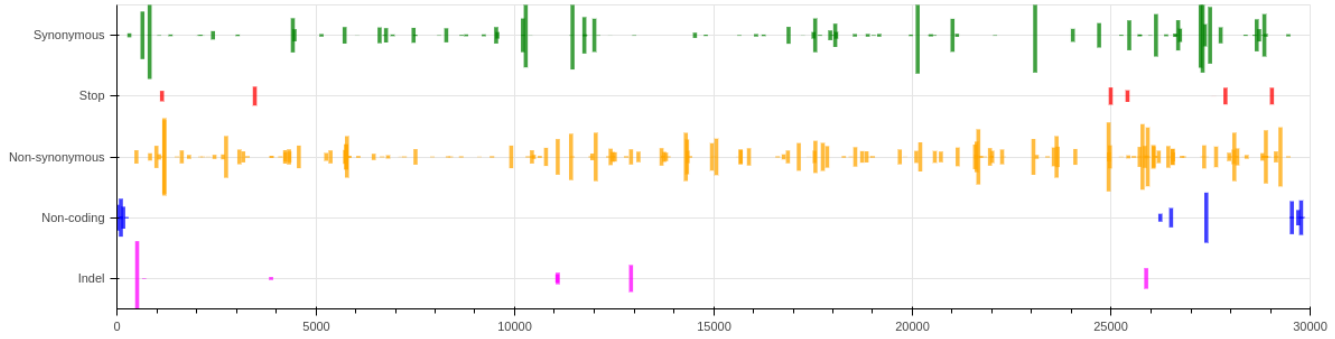

"COG-Pre" (https://covid19.galaxyproject.org/genomics/interactive_images/genomic_dist_cog-pre.html)

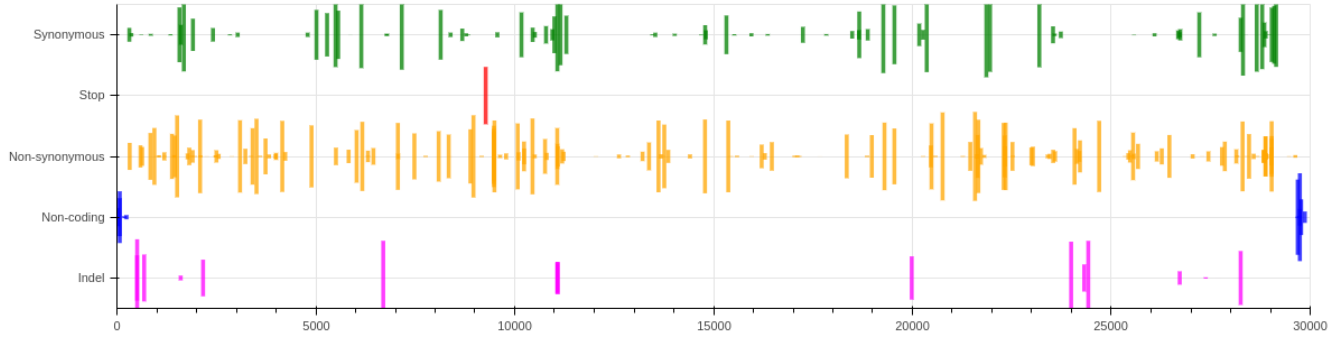

"COG-Post" (https://covid19.galaxyproject.org/genomics/interactive_images/genomic_dist_cog-post.html)

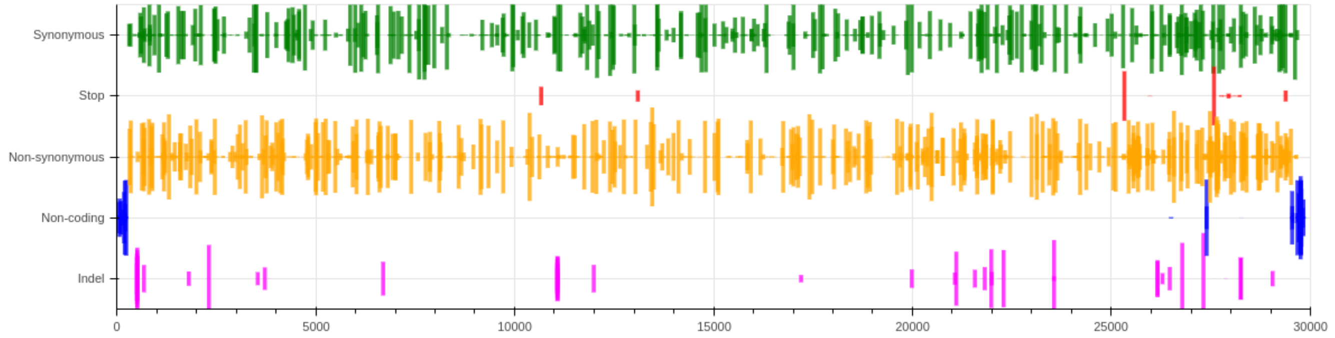

bioRxiv preprint doi: https://doi.org/10.1101/2021.03.25.437046; this version posted March 25, 2021. The copyright holder for this preprint (which was not certified by peer review) is the author/funder, who has granted bioRxiv a license to display the preprint in perpetuity. It is made available under aCC-BY 4.0 International license.

Figure S6. Allelic variant counts per gene.

"Boston" (https://covid19.galaxyproject.org/genomics/interactive_images/gene_dist_boston.html)

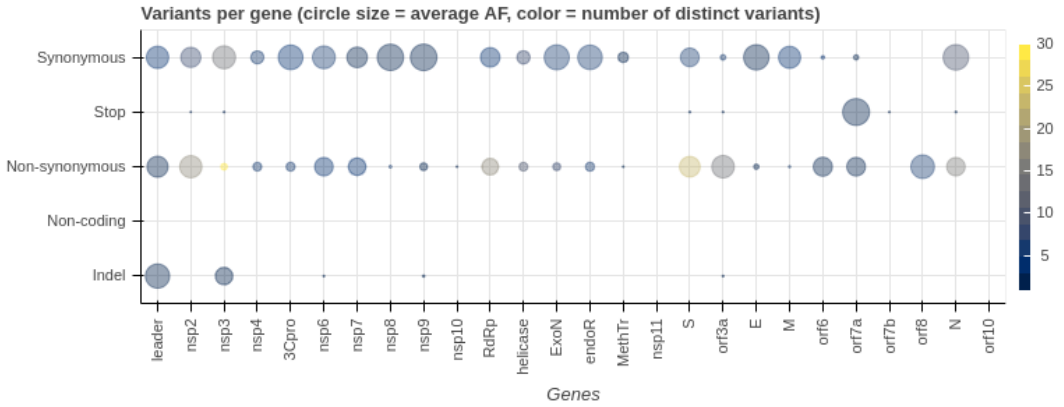

"COG-Pre" (https://covid19.galaxyproject.org/genomics/interactive_images/gene_dist_cog-pre.html)

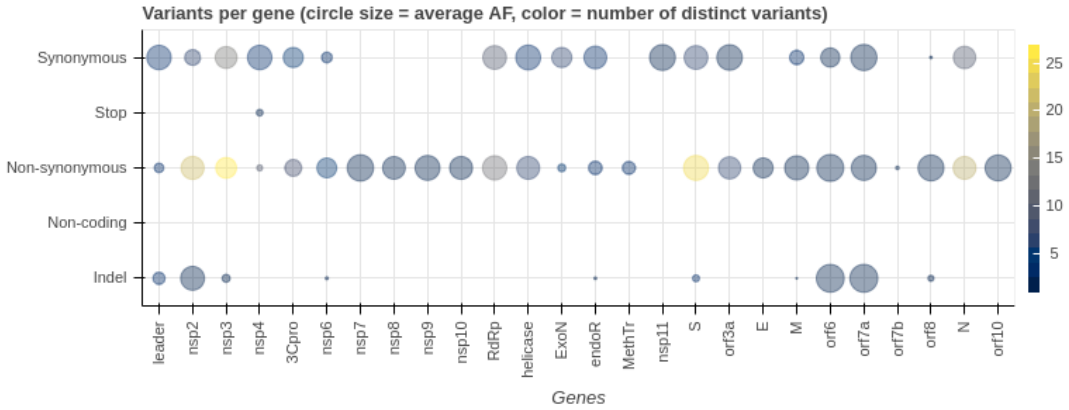

"COG-Post" (https://covid19.galaxyproject.org/genomics/interactive_images/gene_dist_cog-post.html)

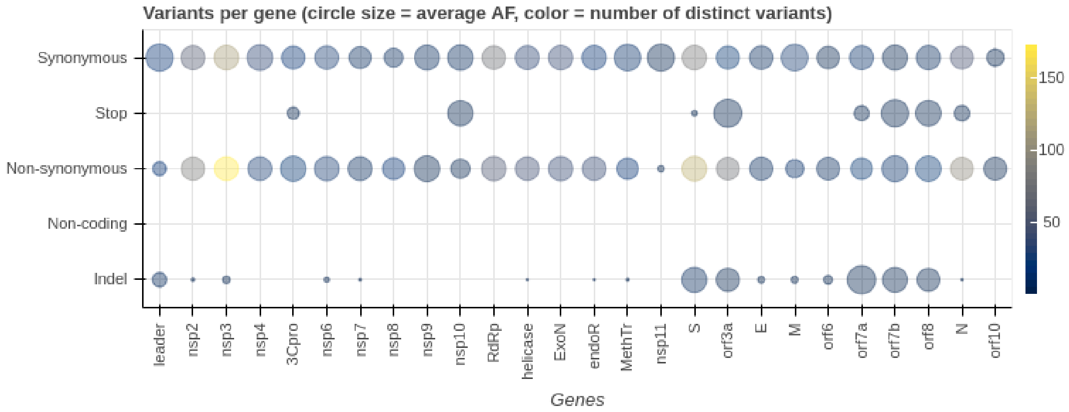

bioRxiv preprint doi: https://doi.org/10.1101/2021.03.25.437046; this version posted March 25, 2021. The copyright holder for this preprint (which was not certified by peer review) is the author/funder, who has granted bioRxiv a license to display the preprint in perpetuity. It is made available under aCC-BY 4.0 International license.

Figure S7. Counts of alternative bases at eight variable locations within pBR322.

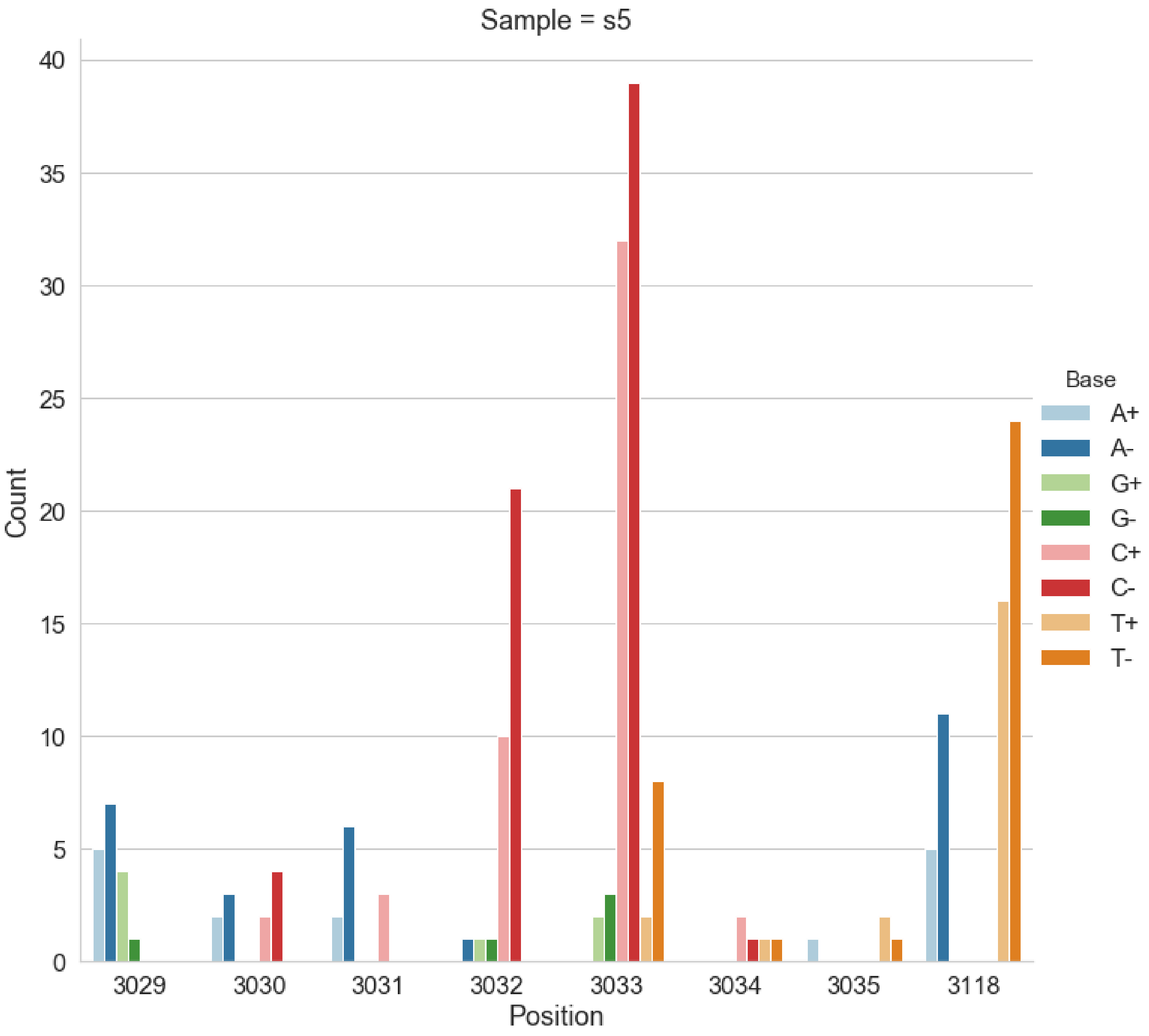

bioRxiv preprint doi: https://doi.org/10.1101/2021.03.25.437046; this version posted March 25, 2021. The copyright holder for this preprint (which was not certified by peer review) is the author/funder, who has granted bioRxiv a license to display the preprint in perpetuity. It is made available under aCC-BY 4.0 International license.

Figure S8. Calls made by `mutect2`, `freebayes`, and `lofreq`. For explanation of x-axis labels see Table S1.

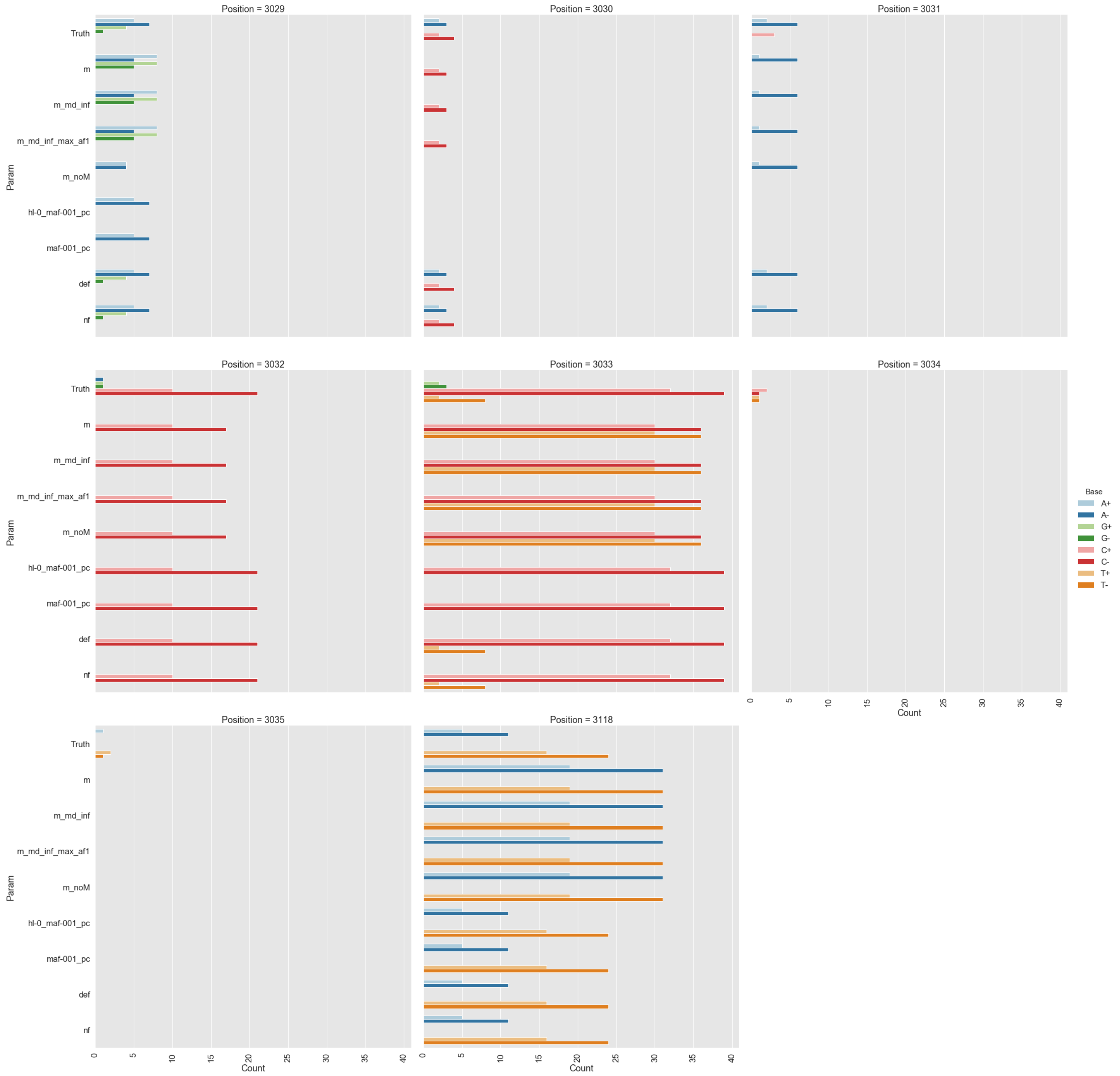

Supplement: 1 [file NIHPP2021.03.25.437046-supplement-1.pdf]
